# Distinct morphological features of NADPH diaphorase neurons across rodent's primary cortices

**DOI:** 10.3389/fncir.2013.00083

**Published:** 2013-04-26

**Authors:** Marco A. M. Freire, José R. Santos

**Affiliations:** ^1^Laboratory of Cellular Neurobiology, Edmond and Lily Safra International Institute for Neuroscience of NatalNatal, Brazil; ^2^Department of Biology, Federal University of SergipeAracaju, Brazil

Nitric oxide (NO) is a versatile gaseous molecule involved in several pathological and physiological functions in nervous system (Calabrese et al., 2007; Steinert et al., 2010; Freire, 2012). Its biosynthesis occurs after the stoichiometric conversion of L-arginine to L-citrulline in a process requiring the presence of NADPH and O_2_ as co-substrates (Bredt and Snyder, 1994). A group of enzymes known as nitric oxide synthases (NOS) is responsible to synthesize NO (Stuehr et al., 2004). In the brain, neuronal NOS is strictly co-localized with NADPH diaphorase (NADPH-d), an oxidative enzyme present in a discrete population of interneurons (Dawson et al., 1991). NADPH-d histochemistry has been used to reveal the presence of NO dispersed in neuropil and to evaluate the presence and distribution of NO-synthesizing neurons across several species (Vincent et al., 1983; Sandell, 1986; Mizukawa et al., 1989; Yan et al., 1996; Franca et al., 2000; Freire et al., 2010, 2011).

Previous studies concerning NADPH-d/NOS reactive neurons focused mainly in its distribution around brain. Differences in morphology were described qualitatively. More recently, a growing amount of information regarding differences in morphometric parameters of this cell group, based in neuronal reconstructions, has emerged (Freire et al., 2007, 2012; Rocha et al., 2012). In light of this, an interesting question arises: could morphological differences of nitrergic neurons among distinct cortical areas or throughout subdivisions of a given area in rodents be associated to their distinct physiologies, as observed to pyramidal neurons (Elston et al., 2006)?

In rodents, primary somatosensory cortex (S1) is a suitable area to be explored since the complete body's representation delineates a topologically correlated cortical map (Rocha et al., 2007; Santiago et al., 2007) commonly revealed by NADPH-d histochemistry (Freire et al., 2012). The general pattern of NADPH-d-reactive neuropil is markedly similar to observed with cytochrome oxidase histochemistry, since both are oxidative enzymes involved in the energetic metabolism (Wong-Riley et al., 1998). The main representation of S1 in rats and mice corresponds to the posterior medial barrel subfield (PMBSF), the region where mystacial vibrissae are represented and the largest and more defined barrels are found. Previous studies have shown that, in a tangential view, NADPH-d neurons concentrate along the regions of PMBSF that separate barrels from each other, known as septa (Franca and Volchan, 1995; Freire et al., 2004). Nevertheless, other S1 regions were not explored in these studies.

In this context, a comprehensive work published by Nogueira-Campos et al. (2012) in Frontiers in Neural Circuits contributes to cover this gap. In that study the authors evaluated the entire population of NADPH-d neurons found across rat's S1 in order to quantify the distribution of these cells throughout all body's representation and also evaluate the morphological aspects and relationship of these neurons with barrels and septal regions. Similar to previously described to PMBSF (Franca and Volchan, 1995; Freire et al., 2004), NADPH-d neurons were not limited by compartment borders across S1. Besides, cell density in septal regions was higher than inside barrels. Regarding morphometric measurements, only in one case evaluated by the authors there was an evident difference between neurons found in septa and barrels, as previously reported (Franca and Volchan, 1995; Freire et al., 2004), what can be explained by the plan of section adopted (coronal *vs.* tangential). But what might these morphological differences represent, in terms of physiology, along distinct regions across rodent's S1? Septal and barrel regions are physiologically/functionally distinct (Alloway, 2008) since septal neurons display significant differences in response latencies related to variations in the frequency of whisker stimulation when compared to barrel neurons (Chakrabarti and Alloway, 2009). In addition both regions receive projections from separated thalamic nuclei (Killackey and Sherman, 2003). Accordingly, barrels and septa would require a segregated circuitry in these different zones, what may be correlated to morphometric differences observed in NADPH-d neurons in both regions. These structural differences could reflect specializations in the processing of sensory information across distinct cortical areas, meaning a difference in their capacity for synaptic integration because dendritic coverage is directly related to the amount of synaptic contacts a cell may receive. Accordingly, neurons possessing a smaller dendritic arbor cover a small cortical area and potentially establish fewer synaptic contacts than more ramified cells. Because septal region is thought to be a segregated cortex from barrels (Killackey and Sherman, 2003), both regions represent two different processing streams that analyze distinct information (Alloway, 2008). So, more complex interneurons in septa could integrate a broader range of synaptic inputs than those found inside barrels.

In conclusion, data provided by Nogueira-Campos et al. (2012) offer new insights about the organization of interneuron circuitry across rat's S1. A further quantification of NADPH-d neuronal morphology in non-primary/association areas may contribute to a more complete notion of cortical differences in the inhibitory circuitry.
